# Resenha sobre o livro *Políticas do Feminino: Saúde, Sexo, Gênero*

**DOI:** 10.1590/0102-311XPT223524

**Published:** 2025-02-24

**Authors:** Laiz Bueno Rodrigues

**Affiliations:** 1 Instituto de Estudos em Saúde Coletiva, Universidade Federal do Rio de Janeiro, Rio de Janeiro, Brasil.

O livro *Políticas do Feminino: Saúde, Sexo, Gênero* ^1^, de Hevelyn Rosa, publicado pela Editora Fiocruz em 2023, na coleção *Criança, Mulher e Saúde*, oferece uma análise crítica das políticas públicas de saúde voltadas para as mulheres no Brasil. Rosa é psicóloga, doutora em Saúde Pública pela Universidade de São Paulo (USP) e está atualmente em pós-doutorado na Faculdade de Saúde Pública (FSP/USP), tendo o livro resultado de sua tese de doutorado defendida em 2021. Utilizando uma abordagem genealógica da História e Antropologia dos documentos, busca caracterizar as definições de “ser mulher” nas políticas de saúde, em especial, na Política Nacional de Atenção Integral à Saúde da Mulher (PNAISM), de 2004, e na Rede Cegonha, de 2011. A partir de uma análise documental detalhada, Rosa questiona a universalidade da categoria “mulher”, desnaturalizando-a e, desse modo, oferecendo uma crítica às implicações dessas normativas. A obra explora como essas políticas perpetuam uma visão cisheteronormativa, limitando a inclusão de grupos que não se enquadram nos padrões tradicionais de feminilidade.

Na Parte I, a autora introduz suas bases teórico-metodológicas. Argumenta que as políticas reiteram a identidade feminina como uma categoria fixa e universal, o que exclui (ou sub-representa) mulheres trans, travestis, lésbicas, prostitutas, negras, indígenas, com deficiência, idosas e outras identidades que não se alinham à visão tradicional de feminilidade. Nesse sentido, Rosa menciona o “peso da reprodução” para mostrar que as questões reprodutivas historicamente são o lócus de ações direcionadas à “saúde da mulher”. Esse tratamento reducionista reforça desigualdades ao impor uma ideia limitada de ser mulher, negligenciando as múltiplas experiências femininas. Podemos relacionar o “peso da reprodução” de Rosa ao “rasgo da reprodução” de Fernandes ^2^ enquanto analogias que evidenciam opressões - iniquidades, precariedades e barreiras - e mobilizam agências no âmbito da saúde sexual e reprodutiva das mulheres e das pessoas que gestam.

Na Parte II, Rosa examina a PNAISM, ressaltando a complexa articulação entre movimentos de mulheres e feministas e o Estado para deslocar as ações de saúde da mulher do âmbito programático para o *status* de política nacional. Rosa aponta que a “*convivência entre diversas perspectivas sobre o que é mulher atravessa a PNAISM como um todo*” (p. 183). A política propõe uma ampliação do conceito de saúde da mulher para além da reprodução, incorporando princípios como integralidade e equidade e temas de saúde como envelhecimento e saúde mental. O modelo hegemônico materno-infantilista de atenção à saúde foi problematizado pelas múltiplas e heterogêneas abordagens dos movimentos sociais que contribuíram significativamente na construção dessa política. No entanto, a autora destaca como o “uso massivo” de conceitos que associam o potencial reprodutivo à identidade feminina, tais como “idade fértil”, “mortalidade materna” e “climatério”, estrutura o documento. Ao dividir a vida da mulher em fases centradas na fertilidade, a PNAISM reproduz uma visão limitada de saúde, restringindo as demandas de grupos marginalizados.

Mello et al. ^3^ estudaram como políticas voltadas à “população LGBT” no Brasil têm enfrentado desafios para alcançar os princípios de universalidade, integralidade e equidade. Segundo os autores, as políticas públicas frequentemente falham em se adaptar às necessidades específicas dessa população, resultando em ações fragmentadas e insuficientes para garantir um atendimento de qualidade, inclusivo e livre de discriminação.

Na Parte III, Rosa examina criticamente a Rede Cegonha, ressaltando suas limitações conceituais e operacionais ao abordar a saúde da mulher. Destaca a ausência da participação dos movimentos feministas e de mulheres em sua formulação. O distanciamento em relação à PNAISM é intencional e evidente. Rosa enfatiza que a Rede Cegonha prioriza eventos específicos - como gravidez e parto - com foco no “planejamento reprodutivo” e no “ciclo gravídico-puerperal”. Além disso, embora a Rede Cegonha aborde temas como planejamento reprodutivo, saúde sexual e educação em saúde, não aloca nenhum recurso específico para essas ações. Com isso, ocupou parte considerável da agenda e “esvaziou” a PNAISM, ao mesmo tempo que promoveu um retrocesso ao adotar o “paradigma materno-infantilista” que une saúde da mulher e infantil em um só conjunto. Segundo Rosa, esse retrocesso reflete a agenda conservadora que privilegia a “proteção da família”, desconsidera o recorte racial-étnico na saúde e se afasta do princípio de integralidade e das demandas feministas, resultando em uma política que não atende à complexidade das necessidades das mulheres brasileiras.

Na Parte IV, Rosa aprofunda a análise das políticas de saúde dos anos 2000, explorando a construção da categoria “mulher” e as implicações do engajamento dos movimentos sociais com o Estado. Ela examina como as políticas públicas continuam a reproduzir uma visão essencialista e cisheteronormativa de “mulher”, associando-a majoritariamente à capacidade reprodutiva e à maternidade. Ela demonstra como, por um lado, os movimentos feministas e de mulheres ampliaram o reconhecimento de direitos e pautas no campo da saúde. Por outro, o Estado frequentemente assimila essas demandas de forma limitada, neutralizando aspectos mais transformadores e reproduzindo desigualdades de gênero, raça e classe. A autora expõe o caráter ambivalente desse engajamento, mostrando que, enquanto é essencial para avanços, também implica concessões e adaptações aos moldes estatais, que priorizam agendas conservadoras em momentos de retrocesso político. Rosa afirma que essas interações são arenas de disputa em que se negocia não apenas o que é “mulher”, mas também quais vidas e experiências são legitimadas ou invisibilizadas pelas políticas de saúde.

Nas *Considerações Finais*, a autora sintetiza sua análise crítica, destacando o caráter dinâmico, conflituoso e repleto de disputas do processo de definição do sujeito “mulher” no campo da saúde brasileira. Ela ressalta que essas políticas operam como “tecnologias de governo” que não apenas regulam corpos e populações, mas também refletem e reproduzem hierarquias de sexo-gênero, articuladas a dimensões econômicas, sociais e políticas. Segundo Rosa, “*não há meios de uma pessoa sem o conjunto feminino de órgãos reprodutores alcançar uma cobertura de cidadania sob o registro da mulher no âmbito da saúde*” (p. 347). Esse silenciamento não é um mero esquecimento, mas uma “*reiteração de um processo hierarquizante no qual se define quem conta como mulher no que concerne ao direito à saúde*” (p. 352), reforçando exclusões estruturais que marginalizam mulheres cujas identidades são subalternizadas.

Arruzza et al. ^4^ também enfatizam que os feminismos devem ser inclusivos e lutar contra todas as formas de opressão, não apenas as que afetam certos tipos de mulheres. Defendem políticas que sejam verdadeiramente interseccionais, capazes de atender às necessidades de todas as “mulheres” e de reconhecer que a exclusão de certos grupos é perpetuada pela influência de estruturas sociais, políticas e econômicas amplas.

A obra de Hevelyn Rosa se destaca pela profundidade analítica e pela inovação metodológica, articulando abordagens genealógicas e referências transdisciplinares. Sua crítica ao binarismo de sexo-gênero e à centralidade da reprodução revela as limitações das políticas analisadas, bem como as dinâmicas de exclusão que perpetuam desigualdades. Rosa conclui com um chamado à utilização de novos paradigmas, como o da justiça reprodutiva, que rompam com as cercas simbólicas que subordinam as mulheres à reprodução e que permitam subverter hierarquias de corpos, sexualidades e arranjos familiares. Para a autora, a Saúde Coletiva deve abraçar perspectivas transdisciplinares que inspirem “*devires, derivações, subversões*” (p. 396) e a construção de vidas mais vivíveis nos interstícios das estruturas opressoras que ainda persistem.

Em síntese, *Políticas do Feminino: Saúde, Sexo, Gênero* é uma leitura indispensável para acadêmicos, profissionais e militantes interessados na interseção entre gênero, saúde e política pública. Hevelyn Rosa não apenas problematiza a produção de normatividades nas políticas de saúde, mas também aponta caminhos para uma transformação paradigmática que valorize a diversidade e a justiça social. Suas contribuições enriquecem significativamente os debates nos campos da Saúde Coletiva e dos Estudos de Gênero, desafiando normatividades e incentivando práticas inclusivas. A partir de sua análise crítica, fica evidente a urgência de romper com as estruturas que subordinam mulheres a papéis reprodutivos, propondo políticas que considerem a integralidade e a interseccionalidade como princípios fundamentais. A obra é um convite à ação e à reflexão, incitando leitores a se engajarem na construção de uma Saúde Coletiva mais equitativa e acessível para todas as mulheres. Recomenda-se vivamente esse livro para aqueles que buscam questionar certezas, desestabilizar normas e vislumbrar novos horizontes para as políticas de saúde sexual e reprodutiva.
